# Do male and female trauma patients receive the same prehospital care?: an observational follow-up study

**DOI:** 10.1186/s12873-016-0070-9

**Published:** 2016-01-19

**Authors:** Rebecka Rubenson Wahlin, Sari Ponzer, Hanna Lövbrand, Markus Skrivfars, Hans Morten Lossius, Maaret Castrén

**Affiliations:** Department of Clinical Science and Education, Södersjukhuset, Karolinska Institutet, SE-118 83 Stockholm, Sweden; Division of Intensive Care Medicine, Department of Anesthesiology, Intensive Care and Pain Medicine, University of Helsinki and Helsinki University Hospital, Helsinki, Finland; Field of Prehospital Critical Care, Network for Medical Sciences, University of Stavanger, Kjell Arholmsgate 41, NO-4036 Stavanger, Norway; Department of Emergency Medicine and Services, University of Helsinki and Helsinki University Hospital, Helsinki, Finland; Department of Anesthesia and Intensive Care, Södersjukhuset, SE-118 83 Stockholm, Sweden

**Keywords:** Gender, Injury, Late adolescent and adult trauma care, Prehospital care, Emergency services

## Abstract

**Background:**

Trauma-related mortality can be lowered by efficient prehospital care. Less is known about whether gender influences the prehospital trauma care provided. The aim of this study was to explore gender-related differences in prehospital trauma care of severely injured trauma patients, with a special focus on triage, transportation, and interventions.

**Methods:**

We performed a retrospective observational study based on local trauma registries and hospital and ambulance records in Stockholm County, Sweden. A total of 383 trauma patients (279 males and 104 females) > 15 years of age with an Injury Severity Score (ISS) of > 15 transported to emergency care hospitals in the Stockholm area were included.

**Results:**

Male patients had a 2.75 higher odds ratio (95 % CI, 1.2–6.2) for receiving the highest prehospital priority compared to females on controlling for injury mechanism and vital signs on scene. No significant difference between genders was detected regarding other aspects of the prehospital care provided.

**Conclusions:**

This study indicated that prehospital prioritization among severely injured late adolescent and adult trauma patients differs between genders. Knowledge of a more diffuse presentation of symptoms in female trauma patients despite severe injury may help to adapt and improve prehospital trauma care for this group.

## Background

Trauma is a major cause of death and permanent disability worldwide [1]. Most trauma-related deaths occur on the scene of the trauma. In European countries, nine prehospital deaths occur for each hospital death of trauma patients aged 65 and younger [2]. Preventable trauma deaths are frequently caused by hemorrhagic shock, a reversible state if treated in time [3]. Prehospital hemorrhage control and initiation of fluid therapy, as well as preventing hypothermia and managing airways, may improve survival [3, 4].

Some studies have suggested differences between genders in terms of type and severity of trauma, the prehospital care provided, and outcome. Wohltmann and colleagues showed that young males have a 27 % higher risk of dying from trauma compared to females [5]. These findings were supported by a Swedish study reporting males to have an increased risk of 1-year mortality even when adjusted for injury severity and other probable confounders [6]. Correct triage and direct transport to a trauma center has been shown to be associated with improved survival [7]. Gomez et al. reported that severely injured females are less likely to be directed to a trauma center [8].

Research on prehospital general trauma care in terms of assessment and treatment on scene and during transportation to hospital is scarce. As far as we know, no studies have been published directly focusing on gender differences of the severely injured trauma patient, although gender aspects have been mentioned. Some studies have been conducted regarding other aspects of the prehospital care. Meisel et al. studied prehospital care of patients presenting with acute chest pain and found that females were less likely to receive aspirin, nitroglycerin, and intravenous access compared to their male counterparts [9]. Furthermore, Kaul et al. reported that women presenting at an emergency department with coronary syndromes were less likely than men to be admitted to an acute care hospital and to be treated with coronary revascularization procedures [10]. In order to ensure gender-equal prehospital care, gender aspects should be integrated in future research [11, 12].

The aim of this study was to explore gender-related differences in prehospital trauma care of severely injured trauma patients, with a special focus on triage, transportation, and interventions.

## Methods

### Study setting

This study was conducted in the area of the Stockholm County Council (SCC), consisting of 26 municipalities covering 6519 km^2^ and including an archipelago of approximately 30,000 islands. Stockholm County has about two million inhabitants, which is about 20 % of the Swedish population [13]. The SCC has the overall responsibility for all healthcare, including the emergency medical services (EMS) and the seven emergency care hospitals, one of which has two sites, but only one of them can be regarded as a level-1 trauma center according to the American College of Surgeons’ criteria [14]. Two private organizations run the prehospital EMS on contracts with the SCC, as well as one organization run by the SCC itself. At the time of the data collection (2008), there were 55 ground ambulances, one helicopter, one mobile intensive care unit (MICU), and three rapid-response vehicles operating in the area. One of the rapid-response vehicles was staffed by an anesthesiologist and the other two by a nurse anesthetist, as well as emergency medical technicians (EMTs). The MICU was staffed by one ambulance nurse and an EMT. The rapid-response vehicle was called in, in addition to a regular ambulance, for severe accidents with the purpose of starting early advanced resuscitation. The helicopter was staffed by a nurse anesthetist, and all regular ground ambulances by EMTs and registered nurses.

**Fig. 1:**
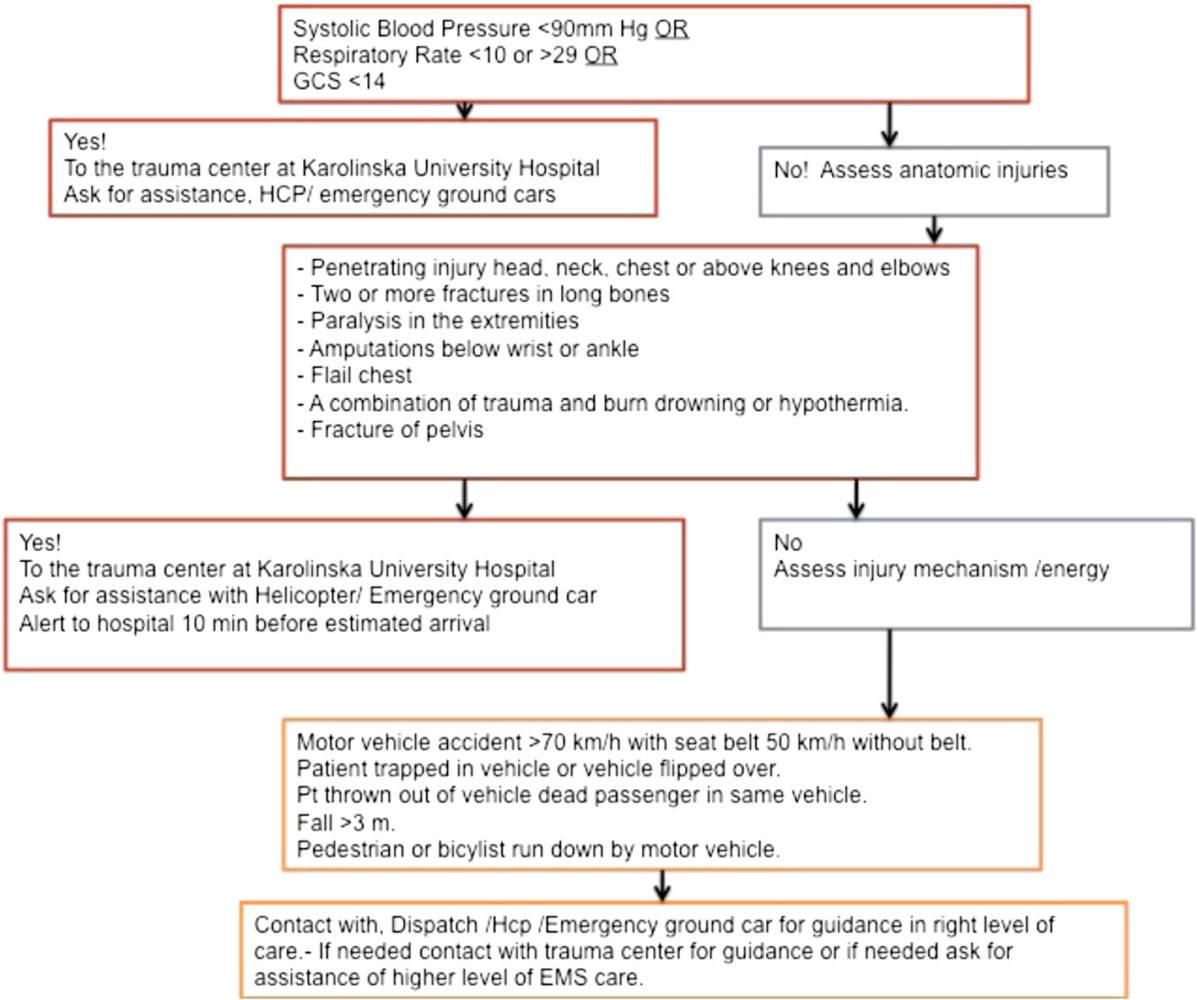
Trauma triage protocol. The trauma triage protocol used in the SCC. The triage protocol also states where to transport the patient. When a patient meets the criteria for transport to a trauma center, the priority is automatically priority 1

The EMS uses three different levels of priority when transporting patients to hospital, with priority 1 being the highest and most urgent level and priority 3 being only transportation. The priority of severe traumas is based on a trauma triage and transport protocol (implemented in 2007) for prehospital use [15] and is derived from ACS-COT field triage criteria [16]. The trauma triage protocol includes vital parameters (i.e., SBP <90, RR <10 or >29, and GCS <14), anatomical injuries, and trauma mechanism. The triage protocol states that if the trauma patient fulfills any of the triage crieria he/she should be transported directly to the trauma center even if it means bypassing a nearer hospital (Fig. 1). Patients who meet the criteria for transport to the trauma center are assigned a level 1 transport priority (i.e., the highest level of prehospital priority) [15].

### Study population

Included were adult and late adolescent trauma patients (>15 years of age) with an Injury Severity Score (ISS) > 15, transported by ground ambulance or helicopter to any of the seven hospitals providing emergency care in the Stockholm area during the period January 1st – December 31st, 2008. Patients with cardiac arrest due to trauma and ongoing cardiopulmonary resuscitation (CPR) during transport to hospital were included even if no return of spontaneous circulation (ROSC) had occurred during transport. Patients declared dead on scene due to trauma and for whom no resuscitative measures were taken, patients admitted to the reporting hospital > 24 h after the trauma, and patients suffering from asphyxia due to drowning were excluded. In addition, we excluded patients transported from another county for specialist care and/or transfers after > 24 h to the university hospital after admission to any of the other hospitals.

Variables recorded were age, gender, predominant type of injury, mechanism of injury, ICD-10 diagnosis, intentional injury, prehospital cardiac arrest, prehospital time intervals, prehospital competence level, i.e., *basic* (EMT and nurse) or *advanced* (nurse anesthetist or anesthesiologist), type of prehospital transportation, airway management, hospital length of stay (LOS), and 30-day mortality, all in accordance with the Utstein trauma template [17]. In addition, the following variables were added for the purpose of this study: prehospital priority (priority 1/other), transport to trauma center (yes/no), administered fluid and analgesics, ISS [18], and the Revised Trauma Score (RTS) [19], including the Glasgow Coma Scale (GCS) [20], systolic blood pressure, respiratory rate, and the 24-h mortality.

Primary outcome measures were prehospital priority and prehospital analgesics given. *Prehospital priority* was chosen since this measure was considered to be a result of the overall prehospital assessment of the patient, in terms of both triage and transport decision. The assessment was based on the trauma triage and transport protocols used in our system (Fig. 1). *Prehospital analgesics* given were chosen as a measure of the prehospital care as seen from a patient perspective.

Secondary outcomes were transport to trauma center, competence level of the prehospital staff, type of prehospital transportation, prehospital airway management, prehospital fluids, prehospital immobilization, 30-day mortality, 24-h mortality, hospital LOS, total prehospital time, and prehospital on-scene time. These outcomes were chosen in order to get a broader view of both the system and the prehospital care.

### Data collection

Data were collected from the trauma registries at Karolinska University Hospital (two sites) (KVITTRA/QUITC, version 14.0) and from Södersjukhuset, a large teaching hospital (TRAUMAREG version TraumaSys 2000–2001, version 1.1.). For Södersjukhuset, the trauma registry data regarding length of stay (LOS) were completed via the hospital’s inpatient digital registration system (Pasett-DRG, version 1.61). Pre-hospital data stemmed from digital ambulance records (CAK-net) used by all ambulance caregivers.

Patients from the four hospitals without trauma registries were identified by a manual search of admission records for each emergency department. Records for all patients with any type of trauma transported by ambulance or helicopter to surgical or orthopedic sections of the emergency departments (EDs), all patients with an ED priority level of 1 or 2 (urgent triage levels), and/or all of those admitted to a hospital ward from the ED were examined. In addition, records of all patients with suspected head trauma or patients directly admitted to the ICU or operating room from the ED were examined regardless of the priority given at the ED. It was not possible to obtain hospital admission records for one of the minor hospitals without trauma registries, and therefore only records for patients reported as “pre-alert” trauma patients were scanned. All patients were identified through the unique personal social security number given to every Swedish citizen. Foreign patients receive a temporary identification number given by the admitting hospital and therefore it was also possible to track these patients. Inpatient data were retrieved via the hospitals’ digital records (Take Care, Melior, and Cambio Cosmic).

The Abbreviated Injury Score (AIS, version 2005) and Injury Severity Score (ISS) [18] were calculated by a trained trauma registrar and by one of the authors (RRW).

### Ethical approval

The study received ethical approval from the Regional Ethical Review Board in Stockholm (Reg. Nos.: 2007/1113-31, 2010/1979-32, 2013/1718-32, and 2014/691-32).

### Statistics

Since none of the background variables showed a normal distribution, medians and interquartile ranges (IQRs) were calculated for continuous variables and, for categorical variables, counts (n) and percent (%) were used. The Mann–Whitney *U*-test was used to calculate continuous data and chi-square for categorical data.

Differences between genders regarding prehospital priority and care were first analyzed using univariable logistic regression. Thereafter, adjusted odds ratios (ORs) with 95 % confidence intervals (CIs) for primary outcomes were derived by multivariable logistic regression analysis, and, for both models, females were used as the reference group. The covariates included in both the univariable and multivariable models were age, predominant type of injury, intentional injury, injury mechanisms, prehospital cardiac arrest, RTS category of the Glasgow Coma Scale, systolic blood pressure, and respiratory rate. Separate analyses of regression models were performed by stratification of the covariates in order to evaluate whether or not gender-based differences were affected by patient or injury characteristics. In these models, each stratification variable was excluded from its respective model. The data analyses followed a similar methodology to that employed by Gomez et al. 2012 in their study on gender-related differences in access to trauma center care [8]. Model calibration was estimated using the Hosmer-Lemeshow statistic and discrimination using the c-statistic. In all models, the c-statistic exceeded 0.85, suggesting excellent discrimination, and the models showed adequate calibration. The software IBM SPSS Statistics, version 22.0.0.0, was used for the analysis. The statistical significance level was set to *p* < 0.05.

## Results

### Background data

During the study period, a total of 383 patients, 279 males (72.8 %) and 104 females (27.2 %) (*p* < 0.001), with an ISS > 15 were included. Table 1 shows the baseline characteristics for all patients. There were no significant differences in the median age between males (median 45 years, IQR 27–64) and females (median 50 years, IQR 29–77) (Table 1).

**Table 1:** Patient characteristics and background factors by gender (n = 383)

Gender	Male	Female	*P* value
	*n* (%)	*n* (%)	* sign.
Patients	279 (72.8)	104 (27.2)	< 0.001*
Age groups			0.050
15–39 years	116 (41.6)	37 (35.6)	
40–64 years	96 (34.4)	29 (27.9)	
≥ 65 years	67 (24.0)	38 (36.5)	
Injury Severity Score (ISS),			0.935
ISS, 15–29	209 (74.9)	76 (73.1)	
ISS, 30–44	50 (17.9)	20 (19.2)	
ISS ≥ 45	20 (7.2)	8 (7.7)	
Predominant type of injury			0.225
Blunt	252 (90.3)	98 (94.2)	
Penetrating	27 (9.7)	6 (5.8)	
Injury mechanism			0.019*
Traffic	113 (40.6)	39 (37.5)	
Low-energy fall	43 (15.5)	28 (26.9)	
High-energy fall	68 (24.5)	27 (26)	
Other	55 (19.7)	10 (9.6)	
Intentional injury			0.041*
Accident	229 (82.7)	87 (84.5)	
Self-inflicted	13 (4.7)	10 (9.7)	
Assault	35 (12.6)	6 (5.8)	
Missing	2	1	
Predominant Anatomical Injury			0.065
Isolated head	54 (19.6)	23 (22.3)	
Head	74 (26.8)	32 (31.1)	
Chest	69 (25.0)	20 (19.4)	
Abdomen	35 (12.7)	8 (7.8)	
Pelvis	10 (3.6)	12 (11.7)	
Spine and Spinal cord	22 (8.0)	5 (5.9)	
Amputated limb or severe injured extremity	6 (2.2)	1 (1.0)	
≥ 2 long bone fractures	6 (2.2)	2 (1.9)	
Missing	3	1	
Systolic blood pressure, RTS category			0.820
Systolic blood pressure, RTS 4	226 (85.0)	84 (84.0)	
Systolic blood pressure, RTS 0–3	40 (15.0)	16 (16.0)	
Missing	13	4	
Respiratory rate, RTS category,			0.951
Respiratory rate, RTS 4	226 (85.6)	85 (82.5)	
Respiratory rate, RTS 0–3	38 (14.4)	14 (14.1)	
Missing	15	5	
Glasgow Coma Scale, RTS category			0.200
Glasgow Coma Scale, RTS 3–4	207 (76.4)	85 (82.5)	
Glasgow Coma Scale, RTS 0–2	64 (23.6)	18 (17.5)	
Prehospital cardiac arrest	12 (4.3)	3 (2.9)	0.538

* is a marker for a significant finding and the p level was set to <0,05

The median ISS did not differ between males (24, IQR 18–30) and females (25, IQR 18–30) (Table 1). The most frequent injury type for both genders was blunt trauma and the predominant injury mechanism was a traffic accident (Table 1). The anatomical injuries did not differ between groups and for both genders the most frequent injury was head injury (Table 1). Female gender was significantly more frequent among patients with self-inflicted injuries, while males were more often exposed to assaults (*p* = 0.041). RTS categories (Glasgow Coma Scale, systolic blood pressure, and respiratory rate) did not differ between genders and neither did the rate of prehospital cardiac arrest (Table 1).

### Outcome data

There was no difference between genders regarding prehospital on-scene time (Table 2). Male patients were significantly more often given priority 1 (*p* < 0.001), were more often transported straight to a trauma center (*p* = 0.016) and were also more often allocated the highest level of prehospital competence (*p* = 0.033) compared to female trauma patients. Type of transportation, prehospital airway management, fluids, analgesics, or immobilization did not differ between genders. The same was true for hospital LOS, as well as for mortality at 24 h or at 30 days (Table 2).

**Table 2:** Outcome variables by gender

Gender	Male	Female	*P* value
	*n* (%)	*n* (%)	* sign.
Prehospital priority			<0.001*
Priority 1	238 (88.1)	72 (72.0)	
Priority > 1	32 (11.9)	28 (28.0)	
Transport to trauma center			0.016*
Yes	232(83.2)	75(72.1)	
No	47(16.8)	29(27.9)	
Highest level of prehospital competence			0.033*
Basic	105(38.7)	52(51.0)	
Advanced	166(61.3)	50(49.0)	
Type of prehospital transportation			0.457
Ground ambulance	208(76.8)	76(73.1)	
Helicopter	63(23.2)	28(26.9)	
Prehospital airway management			0.721
Not intubated	248 (89.9)	92 (91.1)	
Intubated	28 (10.1)	9 (8.9)	
Prehospital fluids			0.077
No fluids	177 (63.4)	76 (73.1)	
Fluids	102 (36.6)	28 (26.9)	
Prehospital analgesics			0.611
No analgesics	191 (68.5)	74 (71.2)	
Analgesics	88 (31.5)	30 (28.8)	
Prehospital immobilization of neck and spine			0.105
No immobilization of both neck and spine	131 (47.0)	58 (56.3)	
Immobilization of both neck and spine	148 (53.0)	45 (43.7)	
30-day mortality (yes)	51 (18.5)	17 (17)	0.731
24-h mortality (yes)	31 (11.4)	11 (11.2)	0.972
Hospital LOS, median days (IQR)	9 (4–20)	8 (3–16)	0.222
Total prehospital time, median min. (IQR)	42 (32–53)	43 (34–52)	0.572
Prehospital on-scene time, median min. (IQR)	17 (12–24)	17 (12–22)	0.496

* is a marker for a significant finding and the p level was set to <0,05

### Injury mechanism within the blunt trauma group

Blunt trauma was the most common injury type (Table 1) for both genders. Within the blunt trauma group, the most common trauma mechanism was traffic-related injury for both genders, but the second most common mechanism differed between genders. For women, it was low-energy falls and, for men high-energy falls (*p* = 0.019). On stratifying age and trauma mechanism, the most common injury mechanism in the age group ≥65 was low energy falls for both genders. Low energy falls accounted for 77.8 % of the cases involving women 65 years of age or older and, among men in the same age group, low-energy falls accounted for 66.7 % of the trauma cases (Table 3).

**Table 3:** Blunt trauma, injury mechanism by age groups and by gender, n and (%)

Gender	Injury mechanism category				
	Age groups	Traffic-related	Low-energy falls	High-energy falls	Other
Male	15–39	60 (53.1)	3 (7.1)	21 (31.8)	14 (45.2)
	40–64	36 (31.9)	11 (26.2)	27 (40.9)	16 (51.6)
	= > 65	17 (15.0)	28 (66.7)	18 (27.3)	1 (3.2)
Female	15–39	17 (44.7)	2 (7.4)	12 (44.4)	4 (66.7)
	40–64	12 (31.6)	4 (14.8)	8 (29.6)	2 (33.3)
	= > 65	9 (23.7)	21 (77.8)	7 (25.9)	0 (0.0)

### Logistic regression analyses

The univariable logistic regression analysis showed an OR of 2.89 (95 % CI, 1.6–5.1; *p* < 0.001) for male patients to receive the highest priority, compared to females. After adjusting for age, predominant type of injury, intentional injury, injury mechanisms, prehospital cardiac arrest, and RTS, the OR was 2.75 (95 % CI, 1.2–6.2; *p* = 0.015). No interactions were found between the patients’ gender and the variables adjusted for.

**Fig. 2:**
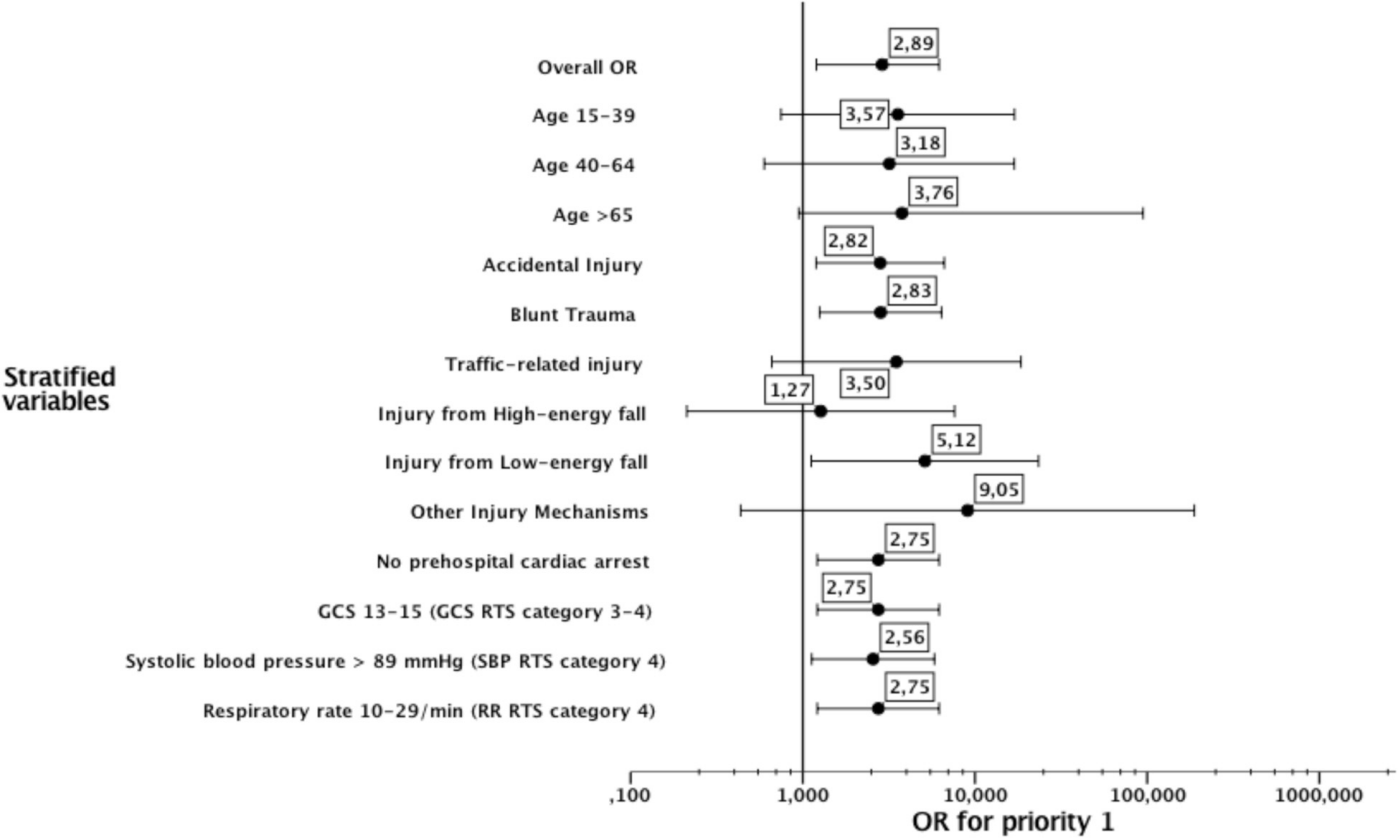
Prehospital Priority. Prehospital priority-adjusted ORs and 95 % CIs for male and female patients, stratified by patient and injury characteristics, as listed in Table 1. Every stratified variable was not included in each of the multivariable models. For the variables penetrating injury, assault, and self-inflicted injury, the number of patients was too small to be included in the analyses. The vital signs were categorized as Revised Trauma Score (RTS) categories; Glascow Coma Scale (GCS) systolic blood pressure (SBP), and respiratory rate (RR)

When the analyses were stratified and adjusted for the association between the highest prehospital priority and male gender, the likelihood of a higher priority was relatively the same over strata (Fig. 2). The exceptions were the injury mechanism categories, “Low-energy fall”, OR 5.12 (95 % CI, 1.1–23.4) and “Other”, OR 9.05 (95 % CI, 0.44–187.9), which showed an increased likelihood of a higher priority for males. In addition, the injury mechanism “high-energy fall” showed a lower likelihood OR of 1.27 (95 % CI, 0.21–7.62) of receiving the highest priority for males.

There was no difference between genders regarding prehospital-administered analgesics (31.5 % and 28.8 %, respectively; *p* = 0.611) and the multivariable logistic regression analysis did not show any significant gender differences regarding the likelihood of either gender to receive analgesics (OR_adj_ for males, 0.86; 95 % CI, 0.49–1.51; *p* = 0.609). However, there was an increased likelihood for the age group 15–39 years (OR, 2.11; 95 % CI, 1.02–4.37; *p* = 0.044) to receive analgesics. The analysis also revealed a lesser likelihood for patients with a systolic blood pressure below 90 mmHg to receive prehospital analgesics (OR, 0.4; 95 % CI, 0.17–0.87; *p* = 0.022), as well as a lesser likelihood of receiving analgesics if the injury mechanism was a low-energy fall (OR, 0.15; 95 % CI, 0.04–0.66). No interactions were found between gender and age group 15–39 years (*p* = 0.728), systolic blood pressure below 90 mmHg (*p* = 0.891), or a low-energy fall (*p* = 0.732).

## Discussion

The aim of this study was to explore gender-related differences in prehospital trauma care of severely injured trauma patients with a special focus on triage, transportation, and prehospital interventions. The main finding was that female trauma patients were less likely to be given the highest prehospital priority, the highest prehospital competence level, and direct transport to the designated trauma center. We did not, however, find any differences between the genders regarding administered prehospital interventions, LOS at hospital, or other outcomes.

Our results point towards a gender-related difference in prehospital assessments of the severity and handling of trauma patients, which is in line with a study by Chang et al. [21] focusing on undertriage in an elderly population (>65 years). In a subanalysis of their main work, they also reported on transport to a designated trauma center of priority-1 patients who met the ACS criteria. The results of this subanalysis showed that males were significantly more likely (OR 1.36) to be transported to a trauma center than females*.* The authors did not further explore this finding, but it is of interest since their population consisted of individuals over 65 years of age. Hsia et al. [22] demonstrated a similar finding to that of Chang et al. in their study on trauma center use among elderly patients and showed that female gender entailed a lesser likelihood of being admitted to a trauma center. Gomez et al. [8] also demonstrated a lower likelihood for females to be admitted to a trauma center after adjusting for other factors, such as age, injury severity, type of prehospital provider, and mechanism of injury. These studies, despite different aims, suggest that also in an elderly population, males are more likely to be transported to a trauma center.

We found differences in trauma mechanism between genders, namely, that the second most common trauma mechanism for females was a low-energy fall (26.9 %). On stratifying for age and trauma mechanism within the dominant blunt trauma group, the difference was even more pronounced (Table 3). Gomez et al. [8] reported a similar pattern and showed that falls from the same level constituted 41 % of the trauma cases among females in their population, which was also the most common trauma mechanism in their study regardless of age. Perhaps this might be one of the reasons why females, despite severe injury, are not recognized at scene as potential severe trauma patients since the trauma mechanism is considered to be of low energy. In our study, we adjusted for age and trauma mechanism, but still males were more likely to be prioritized higher. However, this might only apply to female patients with normal physiological parameters in cases where the triage protocol does not consider the potential difference between genders in symptom presentation. If the trauma mechanism seems mild, the need for a trauma center transport might not be obvious. This could be one of the reasons for the difference between genders in our study.

Earlier studies focusing on the association between gender and trauma mortality are inconclusive [23]. It has been suggested that different biological features of males and females might impact trauma survival. Some studies argue that estrogens are protective in terms of survival after trauma-related shock. Haider et al. showed that females in the fertile period of life had a 14 % lesser risk of dying from trauma-related shock compared to males [24]. This difference has not been seen when comparing males with pre- and post-hormonal females. Male gender has been shown to be a risk factor for one-year mortality, but not for 30-day mortality in elderly populations [6], while other studies have shown no differences in mortality between genders [25, 26]. We did not have the data for a one-year follow-up, but for 24-h and 30-day mortality, no differences between genders were noted.

The importance of a short on-scene time has been discussed in several studies. Some have reported that helicopter transport and/or intubation might prolong the on-scene times [27, 28] and also the presence of a physician on scene [29]. On the other hand, the latter has also been associated with a more agressive treatment, high-precision triage, and rapid transport to the correct level of care [30]. In this study, we chose to focus on the association between on-scene time and gender and found no differences. However, a significant difference between genders was demonstrated regarding the competence level of the prehospital staff or advanced life support provided on scene (i.e., presence of a nurse anesthetist or anesthesiologist), a finding that we have not found in any other published reports. It is not obvious what these findings represent, and they need further investigation.

Trauma occurs more frequently among males [5, 23, 25, 31, 32], a fact confirmed by this study: 72.8 % of the patients in our study were males. The predominant injury mechanism was traffic-related, which conforms with the fact, that in Sweden, severe trauma is most frequently related to motor-vehicle crashes [33, 34]. Annually, an average of 7100 males (61 %) and 4600 females (39 %) are hospitalized due to motor-vehicle crashes [33]. In 2012, 218 males (76 %) and 67 females (24 %) in Sweden died in motor-vehicle crashes [34], showing a gender difference in mortality rates which is consistent worldwide [11].

On evaluating the on-scene variables, i.e., airway management, administration of intravenous fluids, pain management, and stabilization of neck and spine, no gender differences were evident in our data, which is well in line with the study by Schoeneberg et al. [23]*.* Other studies from the prehospital settings have shown that females reported more pain, but were less likely to receive morphine [35] and that female patients with isolated extremity injuries were less likely to receive analgesics [36]*.* On the other hand, Raftery et al. [37] investigated patients in the ED presenting with headache, neck pain, or back pain and concluded that females were more likely than males to report pain and also to receive more analgesics.

Our triage protocol and transport directives state that if the patient is recognized as priority 1, the patient should be transported directly to the trauma center, but in cases where a patient has a compromised airway, the EMS are allowed to choose a closer non-trauma hospital to secure the airway. This aspect has not been included in our analysis. It is also worth noticing that in the current trauma triage protocol, age is not included in the triage algorithm.

A strengt of this study is that we included all patients during one year in a well-defined area covering about one fifth of Sweden’s population and all patients were traceable via the unique individual Swedish social security number. Nevertheless, the relatively small study sample and the fact that the partient-related outcome was measured only in terms of mortality may limit the validity of our findings.

## Conclusions

In an urban part of Sweden covering one fifth of the Swedish population, we found that female trauma patients were less likely to receive the highest prehospital transport priority and were less likely to be transported directly from the scene to a trauma center. We also found that the trauma mechanism differed between genders, but this did not affect the outcome. Prehospital interventions and other system outcomes did not differ between genders. Recognizing gender differences with educational efforts and in pre-hospital trauma management protocols may expedite the trauma care of female patients.

## Abbreviations

ACOS: American College of Surgeons
AIS: Abbreviated Injury Scale
CPR: Cardiopulmonary resuscitation
ED: Emergency Department
EMS: Emergency Medical Services
EMT: Emergency medical technician
GCS: Glasgow Coma Scale
ICU: Intensive care unit
ISS: Injury Severity Score
KSS: Karolinska University Hospital in Solna
KVITTRA/QUITC: (Quality in Trauma Care)
LOS: Length of stay (days)
ROSC: Return of spontaneous circulation
RTS: Revised Trauma Score
RR: Respiratory rate
SBP: Systolic blood pressure
SCC: Stockholm County Council
